# Louis J. Steele and Charles F. Goldie. The arrival of the Maori in New Zealand (1898).

**DOI:** 10.3201/eid0705.010531

**Published:** 2001

**Authors:** R Blackley


### The Cover

### Louis J. Steele and Charles F. Goldie
### The Arrival of the Maoris in New Zealand (1898)

**Oil on canvas, 1380 mm x 2450 mm, Auckland Art Gallery Toi o Tamaki, bequest of Helen Boyd, 1899.**

The late 19th-century emergence in New Zealand of the history-painting tradition relates to the European settler (Pakeha) culture's artistic and literary quest for a national identity. By the 1880s the larger towns had established museums and art galleries intent on acquiring important "national" works, as well as art societies that promoted the achievements of local painters. Pakeha writers were crafting new accounts of the distant Maori past, interrogating and embellishing traditional migration legends. Inevitably, painters appropriated this legendary past for their own artistic purposes.

The English-born Louis J. Steele (1843-1918) had trained in Paris during the 1860s and had lived through the Prussian siege and Commune of 1870-71. Cutting a calculatedly bohemian figure in 1880s Auckland, Steele became a leading artist and influential teacher. He encouraged his New Zealand-born protegé Charles F. Goldie (1870-1947) to seek further training with the cosmopolitan Académie Julian in Paris, where he studied from 1893 to 1897. Goldie's training involved the copying of famous works from the Louvre, including Théodore Géricault's early 19th-century history painting The Raft of the Medusa–the compositional inspiration for The Arrival of the Maoris.

Goldie and Steele displayed their painting, which was on a monumental scale by colonial standards, first at their studio and subsequently at the 1899 exhibition of the Auckland Society of Arts. Critics hailed its evocation of the moment in which the desperate mariners sighted their new land, and the "gruesome" and "appalling" evidence of the travelers' privations: "There is a terrible attraction in these naked emaciated figures huddled in all different postures of agony and despair in the canoe" (1). Recent scholarship has linked Pakeha enthusiasm for The Arrival of the Maoris to the 19th-century predilection for shipwreck imagery, as well as to the comforting message that the Maori were themselves immigrants (2).

As early as 1902 there was a report that "Maoris who view the picture in the Art Gallery are indignant at the manner in which it is represented that the natives arrived in New Zealand" (3). Another writer in 1934 described Maori elders' responses to The Arrival of the Maoris and a similar work as follows: "Far from being appreciative, they always regard them with dubious feelings and disdain. To them they are mere creations of the Pakeha mind and not consistent with the traditional records of the matters represented" (4). Maori revulsion towards the painting relates to more than the diminishment of Polynesian maritime prowess, or to the many historical inaccuracies, for the depiction of a desperate band hurtling forward on a broken craft represents a graphic realization of the widespread colonial mythology of the "dying race."

Despite Maori dismay and the vigorous denunciation of ethnologists, The Arrival of the Maoris in New Zealand has secured a firm hold on the mechanisms of perpetual fame. It enjoys a more active reproduction cycle than any other New Zealand historical painting, appearing even within Maori publications. The checkered history of its reception suggests that such paintings of "history," especially colonial appropriations of a Maori past, are likely to remain highly contested.

**Roger Blackley**
Victoria University of Wellington
New Zealand

### References

1. Blackley R. Goldie. Auckland: Auckland Art Gallery and David Bateman,1997.
2. Bell L. Life and Death at Sea: L.J. Steele and C.F. Goldie's The Arrival of the Maori in New Zealand, 1898. Bulletin of New Zealand Art History 1974; 3:3-8.
3. Unknown Author. French Academy of Art Exhibition. New Zealand Graphic 1899 Nov 4; 832.
4. Graham G. Maori Customs. Faults in historical pictures. Auckland Star 1934 Aug 3;

**In the next issue of Emerging Infectious Diseases, November-December 2001**

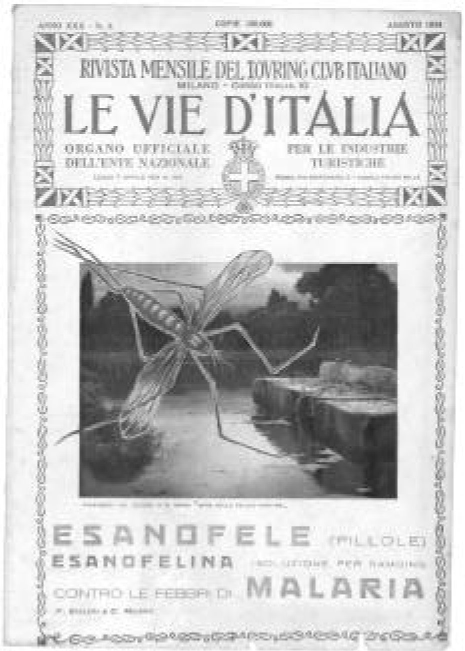

**Could Malaria Reappear in Italy?**

**Other Articles Include**

***Trichomonas vaginalis*, HIV, and African-Americans**

**Advanced Age a Risk Factor for Illness Temporally Associated with Yellow Fever Vaccination**

For a complete list of articles included in the November-December issue, and for articles published online ahead of print publication, see http://www.cdc.gov/ncidod/eid/upcoming-5.htm

